# Multi-analyte proteomic analysis identifies blood-based neuroinflammation, cerebrovascular and synaptic biomarkers in preclinical Alzheimer’s disease

**DOI:** 10.1186/s13024-024-00753-5

**Published:** 2024-10-10

**Authors:** Xuemei Zeng, Tara K. Lafferty, Anuradha Sehrawat, Yijun Chen, Pamela C. L. Ferreira, Bruna Bellaver, Guilherme Povala, M. Ilyas Kamboh, William E. Klunk, Ann D. Cohen, Oscar L. Lopez, Milos D. Ikonomovic, Tharick A. Pascoal, Mary Ganguli, Victor L. Villemagne, Beth E. Snitz, Thomas K. Karikari

**Affiliations:** 1 Department of Psychiatry, School of Medicine, University of Pittsburgh, 3811 O’Hara Street, Pittsburgh, PA 15213 USA; 2 Department of Chemistry, University of Pittsburgh, Pittsburgh, PA 15213 USA; 3 Department of Human Genetics, School of Public Health, University of Pittsburgh, Pittsburgh, PA 15213 USA; 4 Department of Neurology, School of Medicine, University of Pittsburgh, Pittsburgh, PA 15213 USA; 5 Geriatric Research Education and Clinical Center, VA Pittsburgh HS, Pittsburgh, PA USA; 6 Department of Epidemiology, School of Public Health, University of Pittsburgh, Pittsburgh, PA USA

**Keywords:** Preclinical Alzheimer’s disease, Plasma biomarkers, Proteomics, Amyloid pathology, Tau pathology, Neurodegeneration, NUcleic acid-Linked Immuno-Sandwich Assay (NULISA), NULISA with next-generation sequencing readout (NULISAseq)

## Abstract

**Background:**

Blood-based biomarkers are gaining grounds for the detection of Alzheimer’s disease (AD) and related disorders (ADRDs). However, two key obstacles remain: the lack of methods for multi-analyte assessments and the need for biomarkers for related pathophysiological processes like neuroinflammation, vascular, and synaptic dysfunction. A novel proteomic method for pre-selected analytes, based on proximity extension technology, was recently introduced. Referred to as the NULISAseq CNS disease panel, the assay simultaneously measures ~ 120 analytes related to neurodegenerative diseases, including those linked to both core (i.e., tau and amyloid-beta (Aβ)) and non-core AD processes. This study aimed to evaluate the technical and clinical performance of this novel targeted proteomic panel.

**Methods:**

The NULISAseq CNS disease panel was applied to 176 plasma samples from 113 individuals in the MYHAT-NI cohort of predominantly cognitively normal participants from an economically underserved region in southwestern Pennsylvania, USA. Classical AD biomarkers, including p-tau181, p-tau217, p-tau231, GFAP, NEFL, Aβ40, and Aβ42, were independently measured using Single Molecule Array (Simoa) and correlations and diagnostic performances compared. Aβ pathology, tau pathology, and neurodegeneration (AT(N) statuses) were evaluated with [^11^C] PiB PET, [^18^F]AV-1451 PET, and an MRI-based AD-signature composite cortical thickness index, respectively. Linear mixed models were used to examine cross-sectional and Wilcoxon rank sum tests for longitudinal associations between NULISA and neuroimaging-determined AT(N) biomarkers.

**Results:**

NULISA concurrently measured 116 plasma biomarkers with good technical performance (97.2 ± 13.9% targets gave signals above assay limits of detection), and significant correlation with Simoa assays for the classical biomarkers. Cross-sectionally, p-tau217 was the top hit to identify Aβ pathology, with age, sex, and *APOE* genotype-adjusted AUC of 0.930 (95%CI: 0.878–0.983). Fourteen markers were significantly decreased in Aβ-PET + participants, including TIMP3, BDNF, MDH1, and several cytokines. Longitudinally, FGF2, IL4, and IL9 exhibited Aβ PET-dependent yearly increases in Aβ-PET + participants. Novel plasma biomarkers with tau PET-dependent longitudinal changes included proteins associated with neuroinflammation, synaptic function, and cerebrovascular integrity, such as CHIT1, CHI3L1, NPTX1, PGF, PDGFRB, and VEGFA; all previously linked to AD but only reliable when measured in cerebrospinal fluid. The autophagosome cargo protein SQSTM1 exhibited significant association with neurodegeneration after adjusting age, sex, and *APOE* ε4 genotype.

**Conclusions:**

Together, our results demonstrate the feasibility and potential of immunoassay-based multiplexing to provide a comprehensive view of AD-associated proteomic changes, consistent with the recently revised biological and diagnostic framework. Further validation of the identified inflammation, synaptic, and vascular markers will be important for establishing disease state markers in asymptomatic AD.

**Supplementary Information:**

The online version contains supplementary material available at 10.1186/s13024-024-00753-5.

## Background

The recent revision of the amyloid/tau/neurodegeneration (AT(N)) research framework emphasizes that Alzheimer’s disease (AD) is a multifaceted disorder involving diverse brain pathologies and physiological processes [1]. In addition to A (β amyloid deposition), T (pathologic tau), and N (neurodegeneration) categories, which were included in the 2018 update [2], the recent update recommends biomarker assessments for inflammation (I) as well as mixed pathologies such as vascular (V) pathology and synucleinopathy (S). Furthermore, alteration of synapses can occur early in the AD continuum, even before overt neurodegeneration, making the examination of synaptic markers important in preclinical AD [3–5]. This new framework necessitates a diverse set of biomarkers for more accurate diagnosis, prognosis, clinical management, and development/evaluation of therapies. Analyses of multiple biomarkers integrated into a single test can enhance efficiency, reduce analytical errors, and save on specimen volume. However, multi-analyte assays that provide concurrent information on A, T, and N processes are lacking, let alone those that concomitantly include I, V, and S biomarkers. In fact, glial fibrillary acidic protein (GFAP) is the only marker listed under I, while V has no entry in terms of biofluid biomarkers recommended in the revision of the research and diagnostic framework [1].

Previous analyses of cerebrospinal fluid (CSF) implicated associations of several inflammatory, vascular, and synaptic function proteins with amyloid-beta (Aβ) and tau pathologies in AD. Regarding neuroinflammation, the astrocytic protein chitinase-3 like-protein-1 (CHI3L1), also known as YKL-40, has been shown to associate preferentially with tau pathology, while GFAP, a different astrocytic protein, was more strongly linked with Aβ plaque pathology [6–9]. CSF levels of soluble TREM2, a transmembrane receptor protein predominantly expressed by microglia cells, were increased in AD and associated with tau-dependent neurodegeneration and cognitive decline [10–13]. Levels of TREM1, another microglial transmembrane protein, were also shown to increase in AD dementia compared with cognitively unimpaired controls and those with mild cognitive impairment (MCI) [14]. In addition, multiple interleukins (ILs) in CSF were associated with Aβ and tau abnormalities, as well as cognitive decline [15–20]. Similarly, several CSF markers of cerebrovascular integrity, such as soluble platelet-derived growth factor receptor β (PDGFRB), intercellular adhesion molecule 1 (ICAM1), vascular cell adhesion molecule 1 (VCAM1), and vascular endothelial growth factor (VEGFs), synaptic markers including neuronal pentraxin-1 (NPTX1) and neurogranin (NRGN), have been associated with AD and cognitive decline [18, 21–28]. High α-synuclein seed amplification assay positivity has been found in AD and is associated with atypical clinical manifestation [29].

A major challenge in the AD biomarker field is the difficulty in accurately measuring the aforementioned neuroinflammation, cerebrovascular, and synaptic protein markers in blood samples to give reliable performances as shown for their CSF counterparts. The development of blood-based assays for these biomarkers has been greatly impeded by several factors, including interference from the extremely complex blood proteome, low abundance of the target analytes, and signal attenuation by unwanted signal from peripheral sources [30, 31]. For example, assays for synaptic markers including NRGN give good analytical signals in plasma but without the corresponding good biomarker performance as shown in CSF [32, 33].

Recently, a highly multiplexed immunoassay capable of measuring classical AT(N) biomarkers alongside multiple I, V, and S biomarkers in plasma was described [34]. Known as the NULISAseq CNS disease panel, this assay employs an innovative automated technology called NUcleic acid-Linked Immuno-Sandwich Assay (NULISA). Coupling NULISA with next-generation sequencing readout (NULISAseq) allows detection of hundreds of proteins with attomolar sensitivity and an ultra-broad dynamic range [34]. The NULISAseq CNS panel currently consists of ~ 120 protein targets covering the eight pathological hallmarks that define neurodegenerative diseases: namely, pathological protein aggregation, synaptic and neuronal network dysfunction, aberrant proteostasis, cytoskeletal abnormalities, altered energy homeostasis, DNA and RNA defects, inflammation, and neuronal cell death [35].

This study had a three-fold aim. Our first aim was to evaluate the technical performance of the NULISAseq CNS disease panel. For classical AT(N) biomarkers (e.g., p-tau181, p-tau217, p-tau231, Aβ40, Aβ42, GFAP, and neurofilament light chain [NEFL]), we compared the NULISA results with those obtained on the widely used Quanterix Single molecule array (Simoa). The second aim was to examine the diagnostic accuracies and longitudinal profiles of blood-based NULISAseq targets against neuroimaging measures of A, T and N in a population-based cohort of mostly cognitively normal older adults. Thirdly, we aimed to identify novel plasma I, V, and synaptic markers associated with Aβ positron emission tomography (PET), tau PET and magnetic resonance imaging (MRI)-based neurodegeneration measures in the same cohort.

## Methods

### Participants

The Monongahela Youghiogheny Healthy Aging Team-Neuroimaging (MYHAT-NI) is a sub-cohort of the parent MYHAT study, a population-based prospective study of cognitively normal older adults designed to characterize the prevalence of MCI in older adults with a low socioeconomic status in selected Rust Belt regions in southwestern Pennsylvania, USA [36–38]. The MYHAT study recruited participants aged 65 and older via age-stratified random sampling from publicly available voter registration lists. The MYHAT-NI study included a subset of MYHAT participants with a Clinical Dementia Rating (CDR) sum-of-box score [39] of < 1.0 for neuroimaging assessments to investigate the distribution and functional correlates of AD. For this reason, all MYHAT-NI participants had normal (CDR = 0) or only very mildly impaired cognition (CDR = 0.5) at the time of enrollment which started in 2017. The only exclusion criterion was contraindication to neuroimaging. The study had two visits: baseline and approximately two-year follow-up. Sociodemographic information was collected at the baseline visit. Both racial identity and years of education were self-reported. Blood collection, neurophysiological assessment, and neuroimaging, including [^11^C] Pittsburgh Compound B (PiB) PET imaging of Aβ plaques, [^18^F]AV-1451 PET imaging of tau pathology, and structural MRI for neurodegeneration, were performed at both baseline and the follow-up visits following standard protocols [40–42]. Detailed study designs for MYHAT and MYHAT-NI, including participant recruitment strategies, multi-domain cognitive assessments, neuroimaging, and data processing, can be found in previous publications [36, 38]. *APOE* genotyping was determined as previously described [43].

We classified A, T, and N status according to [^11^C] PiB PET, [^18^F]AV-1451 PET, and MRI scans for cortical thickness, respectively. The A status was based on a global [^11^C] PiB standardized uptake value ratio (SUVR) computed by volume-weighted averaging of nine composite regional outcomes (anterior cingulate, posterior cingulate, insula, superior frontal cortex, orbitofrontal cortex, lateral temporal cortex, parietal, precuneus, and ventral striatum) [41]. Participants were classified as A + or A- based on a pre-defined cutoff, with > 1.346 as A +  [44, 45]. For T status, a composite SUVR was computed for each [^18^F]AV-1451 PET by normalizing composite Braak regional values ((Braak I—VI) to FreeSurfer cerebellar gray matter activity [46, 47]. Participants with SUVR > 1.18 were considered T + , <  = 1.18 as T- [48]. N status was based on an AD-signature composite cortical thickness index derived from a surface-area weighted average of the mean cortical thickness of four FreeSurfer regions of interest (ROIs) – entorhinal, inferior temporal, middle temporal, and fusiform – that are most predictive of AD-specific diagnosis and pathology, with < 2.7 as N + [44, 49]. The MYHAT-NI study was approved by the University of Pittsburgh Institutional Review Board (STUDY19020264).

### NULISAseq assay procedures and data processing

Plasma samples were sent to Alamar Biosciences, Inc. for NULISAseq measurements. Alamar Biosciences also provided all the reagents for the NULISAseq assay. The analysis was conducted blinded, with Alamar Biosciences, Inc. unaware of the sample grouping information until after the analysis had been completed. The content of the CNS panel was based on suggestions from multiple experts in CNS disease drug development and biomarker research. It includes both established and emerging biomarkers of neurodegenerative diseases that represent multiple hallmarks of neurodegenerative diseases. For ease of cross-reference, all NULISAseq biomarkers were represented using non-italicized upper-case gene symbols as used by the vendor. Italicized upper-case symbols were used when referring to genes. The complete list of biomarkers with their full protein names and gene symbols included in the NULISAseq panel is provided in Additional file 1.

Plasma samples were thawed and centrifuged at 10,000xg for 10 min to remove particulates. The supernatants were then analyzed using the NULISAseq CNS disease panel on an Alamar ARGO™ prototype system, as previously described [34]. In brief, samples were incubated with a cocktail of paired capture and detection antibodies for the included target protein biomarkers and the internal control (IC). The capture antibodies were conjugated with partially double-stranded DNA containing a poly-A tail and a target-specific barcode, while detection antibodies were conjugated with another partially double-stranded DNA containing a biotin group and a matching target-specific barcode. After incubation, the mixtures underwent magnetic bead-based capture, wash, release, recapture, and a second round of wash processes to purify the formed immunocomplexes. A ligation mix, including T4 DNA ligase and a specific DNA ligator sequence, was utilized to ligate the proximal ends of DNA attached to the paired antibodies, generating DNA reporter molecules containing unique target and sample-specific barcodes. The reporter DNA levels were then quantified by Next-Generation Sequencing (NGS). The plasma samples were randomized in two plates for the assay. Three assay controls were run side-by-side with samples for each plate, including the sample control (2 replicates/plate), the inter-plate control (IPC; 3 replicates/plate), and the negative control (2–3 replicates/plate).

Data normalization was performed to remove potential unwanted technical variation. First, IC-based normalization was done by dividing the target counts for each sample well by that well’s IC counts. IPC normalization was achieved by dividing IC-normalized counts by target-specific medians of the IPC (pooled plasma) sample replicates on that plate. Finally, the data was rescaled and log2-transformed to give a more normal distribution for subsequent statistical analyses. These values are hereafter referred to as NULISA Protein Quantification (NPQ) units. The fold change difference between two groups were calculated as 2^(difference in NPQ)^. The plate-specific limit of detection (LOD) was calculated for each target assay by taking the mean plus three times the standard deviation (SD) of the unlogged normalized counts for the negative control samples on the plate. LODs were then rescaled and log2-transformed as above. Measurements for sample controls were used to evaluate the reproducibility of the assays, including both the within-run and between-run coefficients of variation (CVs).

### Procedures for Simoa assays

Simoa assays were performed on an HD-X instrument (Quanterix, Billerica, MA, USA). Prior to the measurements, plasma samples were thawed at room temperature and centrifuged at 4000xg for 10 min to remove particulates. Plasma NEFL, GFAP, Aβ42 and Aβ40 were measured with the Neurology 4-Plex E (#103670), p-tau181 with the p-tau181 V2 Advantage kit (#103714), and p-tau217 with the ALZpath Simoa® p-Tau 217 V2 Assay Kit (#104371). Quality control (QC) samples of 2–3 different concentrations for each assay were analyzed at the start and the end of each run to assess the reproducibility of each assay. The average within-run CVs of the QC samples were 3.7% for p-tau217, 6.6% for p-tau181, 14.3 for NEFL, 9.9% for GFAP, 8.9% for Aβ42, and 9.5% for Aβ40. The average between-run CVs were 11.4% for p-tau217, 11.7% for p-tau181, 18.3% for NEFL, 17.8% for GFAP, 13.0% for Aβ42, and 14.6% for Aβ40.

### Statistical analysis

All analyses were conducted using MATLAB (version R2021b) or R statistical software version 4.2.1 (R Foundation for Statistical Computing, Vienna, Austria; http://www.r-project.org/). We utilized the Wilcoxon rank-sum test for two-group comparisons and the Kruskal–Wallis test for comparisons involving more than two groups. Spearman's rank correlation was used to measure the strength and direction of association between two continuous variables. For demographic characteristics, continuous variables were presented as median and interquartile range (IQR), while categorical variables were reported as counts. Wilcoxon rank-sum tests and Fisher’s exact tests were employed to assess the significance of differences between A + and A- participants for continuous and categorical variables, respectively. Linear mixed models were used to assess the association of common AD risk factors, including age, sex, and *APOE* ε4 carrier status, with biomarkers.

The following statistical tests were applied to evaluate cross-sectional associations between plasma biomarkers and brain Aβ and tau pathologies: (1) Wilcoxon rank-sum tests for the univariate significance for the associations between NPQs and dichotomous pathology variables (e.g., A- vs. A +), without adjusting for risk factors; (2) Spearman's rank correlation to measure the strength and direction of the associations between NPQs and continuous variables (e.g., Aβ PET SUVR); (3) linear mixed models (random intercepts) with biomarker NPQs as the dependent variable, visit-specific Aβ PET status as the independent variables, as well as common risk factors (such as age, sex and *APOE* ε4 carrier status) to determine the overall risk factor-adjusted significance combining samples from both visits. False discovery rate corresponding to cutoff *p*-values were calculated according to the procedure described by Yoav Benjamini and Yosef Hochberg in 1995 [50]. An arbitrary *p*-value of 0.005 was used as the significance cutoff, which corresponded to 3 to 10% FDR depending on the comparisons. Receiver operating characteristic (ROC) curves and the area under curve (AUC) were calculated using the MATLAB *perfcurve* function, based on scores predicted from generalized linear regression models fitted using the MATLAB *fitglm* function. Confidence intervals were computed using bootstrap with 1000 replicates. DeLong test implemented in the pROC package was used to compare ROC curves [51, 52]. Web app VolcaNoseR was used to draw the volcano plot [53].

Longitudinal analysis was limited to participants with plasma samples analyzed at both visits. We calculated the yearly percentage of change for biomarker NPQs and continuous AD pathology variables using this formula: 100 * ([Follow up – Baseline]/[Baseline]) /Δ Time in years. Wilcoxon rank-sum tests were then used for two-group comparisons, and Spearman's rank correlation to assess the association between yearly plasma biomarker changes and the annual A or T pathology change. Due to the relatively short duration between the two visits, we did not expect drastic changes in both blood and neuroimaging biomarker levels. Therefore, we treated the longitudinal analysis as explorative, and the original rather than FDR-adjusted *p*-values were used to determine significance.

## Results

### Cohort characteristics

This study comprised 176 plasma samples from 113 participants (average age 76.7 years at baseline, 54.0% women, and 95.0% non-Hispanic White) from the MYHAT-NI cohort (see Table 1 for demographic characteristics). These participants were recruited from the parent MYHAT cohort, which includes areas with relatively low socioeconomic status. The median Area Deprivation Index (ADI) in the MYHAT study area is at the 85th percentile nationally, according to the Neighborhood Atlas [54]. Among them, 63 participants (55.8%) provided plasma samples at two visits (baseline and the 2-year visit). At baseline, 85 (75.2%) participants were classified as Aβ-negative (A-) and 28 (24.8%) as Aβ-positive (A +), while 42 (66.7%) and 21 (33.3%) were A- and A + , respectively at the 2-year visit. Regarding tau PET, 74 (65.4%) participants were tau-negative (T-) and 39 (34.5%) as tau-positive (T +) at baseline. At the 2-year visit, 42 (66.7%) participants were T-, and 21 (33.3%) were T + . In terms of neurodegeneration according to cortical thickness, 80 (70.8%) participants were considered N- and 33 (29.2%) N + at baseline, while at the 2-year visit, there were 42 (66.7%) N- and 20 (31.7%) N + participants. One participant had missing N status at the 2-year visit due to poor MRI quality.

**Table 1:** Participant characteristics in the MYHAT-NI cohort

	**MYHAT-NI (Baseline)**				**MYHAT-NI (2-year visit)**			
	**Total**	**Aβ PET –**	**Aβ PET + **	***p-value***^**a**^	**Total**	**Aβ PET –**	**Aβ PET + **	***p-value***^**a**^
**N (%)**	113	85 (75.2%)	28 (24.8%)		63	42 (66.7%)	21 (33.3%)	
**Age (years)**	76.0 (72.0 – 80.3)	75.0 (71.0 – 79.3)	79.0 (73.5 – 82.5)	0.055	77.0 (74.0 – 81.8)	76.0 (74.0 – 81.0)	78.0 (73.8 – 82.3)	0.884
**Sex**				0.275				0.109
**Female (%)**	61	43 (70.5%)	18 (29.5%)		32	18 (56.3%)	14 (43.7%)	
**Male (%)**	52	42 (80.8%)	10 (19.2%)		31	24 (77.4%)	7 (22.6%)	
**Education (years)**	12 (12 – 16)	12 (12 – 16)	13 (12 – 15)	0.583	13 (12 – 16)	14 (12 – 16)	12 (12 – 14)	0.050
**Years between visits**					2.4 (2.2 – 2.6)	2.4 (2.2 – 2.6)	2.4 (2.2 – 2.7)	0.358
**Race**				1.000				1.000
**Non-Hispanic White**	107	80 (74.8%)	27 (25.2%)		60	40 (66.7%)	20 (33.3%)	
**Black/African American**	6	5 (83.3%)	1 (16.7%)		3	2 (66.7%)	1 (33.3%)	
**MMSE**^**b**^				0.430				1.000
> ** = 24**	108	81 (75.0%)	27 (25.0%)		38	26 (68.4%)	12 (31.6%)	
**19—23**	4	3 (7.0)	1 (25.0%)		0	0	0	
**CDR**^**c**^				0.015				0.033
**CDR = 0**	102	80 (78.2%)	22 (21.8%)		54	39 (72.7%1	15 (27.3%)	
**CDR = 0.5**	10	4 (40.0%)	6 (60.0%)		5	(20.0%)	4 (80.0%)	
***APOE* ε4 carrier**				< 0.001				0.014
**Yes**	18	6 (33.3%)	12 (66.7%)		12	4 (33.3%)	8 (66.7%)	
**No**	95	79 (83.2%)	16 (16.8%)		51	38 (74.5%)	13 (25.5%)	
**Tau PET**^**d**^				< 0.001				0.001
**Negative**	74	65 (87.8%)	9 (12.2%)		42	34 (81.0%)	8 (19.0%)	
**Positive**	39	20 (51.3%)	19 (48.7%)		21	8 (38.1%)	13 (61.9%)	
**N Status**^**e**^				0.639				0.395
**Negative**	80	59 (73.8%)	21 (26.3%)		42	26 (61.9%)	16 (38.1%)	
**Positive**	33	26 (78.8%)	7 (21.2%)		20	15 (75.0%)	5 (25.0%)	

The median and interquartile range (IQR) are reported for continuous variables. Frequencies and percentages are shown for categorical variables. ^a^*P*-values were calculated using the Wilcoxon rank-sum test for a continuous variable and Fisher’s exact test for a categorical variable, respectively. ^b^One participant at baseline and 25 at the 2-year visit had missing MMSE assessment. ^c^One participants at baseline and 4 at the 2-year visit missed CDR assessment. ^d^[^18^F]AV-1451 PET, Mean SUVR > 1.18 as positive. ^e^N status is based on cortical thickness, with < 2.7 being positive. One Aβ PET- participant missed MRI for 2-year visit. Both racial identity and education were self-reported

Most participants were cognitively normal at both visits. CDR-based cognitive assessment rated 102 participants (90.2%) as cognitively normal (CDR = 0) and 10 (8.9%) as mildly impaired (CDR = 0.5) at baseline. At the 2-year visit, 54 participants (85.7%) were cognitively normal, and 5 (7.9%) were mildly impaired. One participant at baseline and four at the 2-year visit missed CDR assessments. Similar results were obtained based on Mini-Mental State Examination (MMSE) assessment. Out of 112 participants with MMSE assessment at baseline, 108 (96.4%) participants were cognitively normal (MMSE >  = 24), and 4 (3.6%) were mildly impaired (MMSE between 19 – 23). All participants with MMSE assessment at the 2-year visit (38 out of 63) were cognitively normal.

### Technical performance and head-to-head comparison of the NULISAseq measurements with Simoa assays

**Fig. 1:**
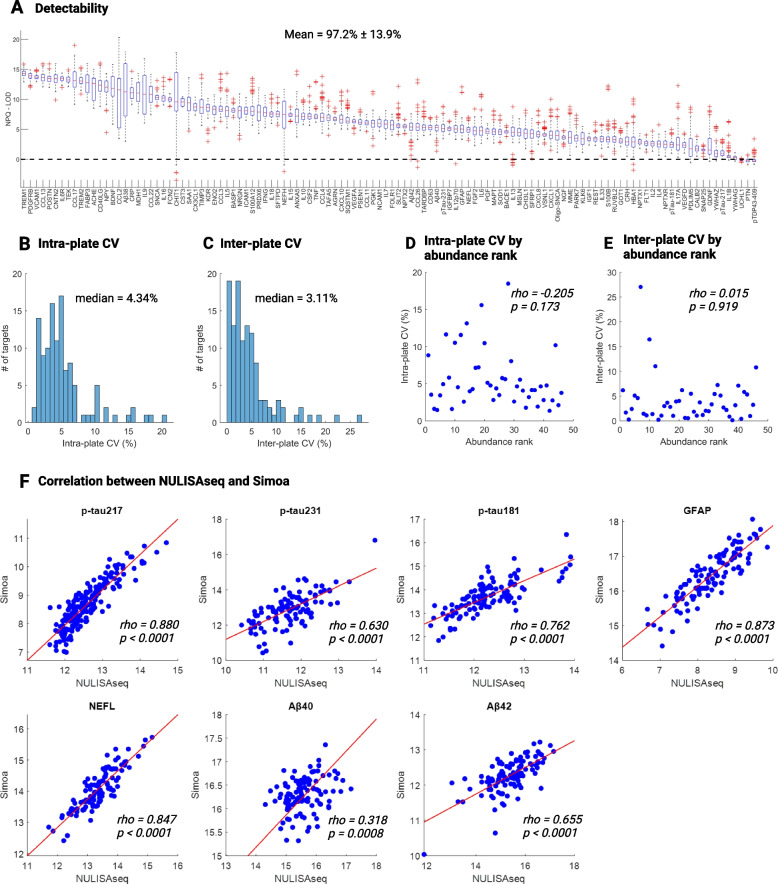
Technical performance of the NULISAseq CNS disease panel. **A** Box plots illustrating the detectability of 116 targets in 176 plasma samples collected from 113 MYHAT-NI participants. The y-axis represents NPQ-LOD, where values > 0 indicate detectability. For each box plot, the central mark indicates the median, and the bottom and top edges of the box indicate the 25th and 75th percentiles, respectively. The whiskers extend to the most extreme data points not considered outliers, and the outliers are plotted individually using the ' + ' marker symbol. Data points were considered outliers if they were greater than q3 + 1.5 × (q3 – q1) or less than q1 – 1.5 × (q3 – q1), where q1 and q3 are the 25th and 75th percentiles of the sample data. **B-C** Histogram distributions of intra-plate (**B**) and inter-plate (**C**) coefficient of variations (CVs). **D-E** Scatterplot distributions between abundance rank and intra-plate (**D**) or inter-plate (**E**) CVs. Intra- and inter-plate CVs were calculated based on results of a pooled plasma sample (SC), measured in duplicates separately in two different plates. Abundance rank was based on the mass spectrometry-estimated protein abundance in the Human Protein Atlas (downloaded on 12/24/2023). **F** Scatterplot distributions illustrating the correlation of protein levels measured using NULISAseq and Simoa method. *Rho* and *p* values were determined using Spearman rank-based correlation. Purple lines indicated the least square regression lines. Abbreviations: NPQ, NULISA Protein Quantification, represents the log2-transformation of normalized target counts; LOD, limit of detection. Simoa measured concentration (fg/ml) was also log2-transformed for this analysis

A total of 116 target assays were incorporated in the NULISAseq CNS disease panel for this study. The plasma concentration range of these targets spanned a minimum of 6 orders of magnitude according to the concentration estimated by mass spectrometry-based proteomics in the Human Protein Atlas database [55, 56]. Despite the broad dynamic ranges of the protein targets, the vast majority exhibited very high detectability, defined as the percentage of samples above the LOD, with a mean ± SD detectability of 97.2% ± 13.9% (Fig. 1A). Only three targets – UCHL1 (ubiquitin C-terminal hydrolase L1), PTN (pleiotrophin), and pTDP43-409 (transactive response DNA binding protein of 43 kDa [TDP43] phosphorylated at Ser409) – had detectability below 70%. The median intra-plate and inter-plate CVs were 4.34% (IQR: 2.80%-6.04%) and 3.11% (IQR: 1.41% -5.45%), respectively, suggesting robust assay reproducibility (Fig. 1B and C). Only two targets – CNTN2 (contactin 2) and NEFH (neurofilament heavy chain) – had inter-plate CVs greater than 20%, a cutoff commonly used for in vitro diagnostic assays. To assess whether the variation depended on protein abundance, we evaluated the association between the intra- or inter-plate CVs and the abundance ranks for targets (n = 46) with plasma concentration data available from the Human Protein Atlas database. As depicted in Fig. 1D and E, both intra- and inter-plate CVs were not influenced by protein abundance, with *p*-values for Spearman rank correlations being 0.173 and 0.919, respectively.

We next examined the correlation between NULISAseq measurements and Simoa measurements of selected biomarkers. These included p-tau217, p-tau231, p-tau181, GFAP, NEFL, Aβ40, and Aβ42. Notably, strong correlations were observed in all pairwise comparisons, with Spearman rank correlation coefficient (*rho*) values spanning from 0.318 to 0.880 (Fig. 1F). P-tau217, GFAP, and NEFL demonstrated the strongest between-platform correlation, with *rho* of 0.880, 0.873, and 0.847, respectively.

To compare the diagnostic accuracies of the two measurements in detecting Aβ PET positivity, we calculated the ROC AUCs using logistic regression models in the baseline samples (Table 2). NULISAseq demonstrated comparable performance to Simoa for all seven biomarkers, irrespective of whether common risk factors (age, *APOE* ε4 carrier status, and sex) were included in the models. For example, at baseline, plasma p-tau217 had AUCs of 0.905 (95% CI: 0.841–0.969) on NULISA and 0.880 (95% CI: 0.800–0.959) on Simoa. When accounting for risk factors, these AUCs increased to 0.930 (95% CI: 0.878–0.980) and 0.925 (95% CI: 0.874–0.977). The DeLong test showed no significant difference between the AUCs. These findings suggest that despite its highly multiplexed nature, the NULISAseq platform performs equivalently as Simoa for quantifying these biomarkers.

**Table 2:** Diagnostic accuracy (ROC analysis) of NULISAseq and Simoa biomarkers for Aβ PET positivity

AUC (95% CI)	ROC AUC with Biomarker only			ROC AUC with Biomarker + risk factors		
	NULISASeq	Simoa	*p-*value	NULISAseq	Simoa	*p-value*
p-tau217	0.905 (0.841–0.969)	0.880 (0.800–0.959)	0.625	0.930 (0.878–0.983)	0.925 (0.874–0.977)	0.891
p-tau231	0.718 (0.615–0.822)	0.632 (0.517–0.746)	0.274	0.806 (0.709–0.903)	0.790 (0.689–0.891)	0.822
p-tau181	0.576 (0.468–0.684)	0.669 (0.560–0.778)	0.237	0.780 (0.678–0.882)	0.793 (0.662–0.893)	0.867
GFAP	0.732 (0.640–0.825)	0.743 (0.653–0.834)	0.867	0.808 (0.717–0.899)	0.816 (0.725–0.908)	0.901
NEFL	0.565 (0.442–0.688)	0.599 (0.480–0.719)	0.695	0.766 (0.655–0.878)	0.791 (0.682–0.901)	0.755
Aβ40	0.539 (0.409–0.669)	0.650 (0.530–0.771)	0.221	0.773 (0.670–0.875)	0.816 (0.719–0.913)	0.547
Aβ42	0.531 (0.411–0.650)	0.550 (0.432–0.667)	0.826	0.779 (0.671–0.886)	0.789 (0.691–0.887)	0.884

Risk factors included age as a numeric variable, sex (male and female), and *APOE* status (*APOE* ε4 carrier or non-carrier). The confidence interval of AUC was computed using the bootstrapping approach. *P*-value was determined using the DeLong test implemented in the pROC package in RStudio

### Association of NULISAseq targets with PET measure of amyloid pathology (A)

#### Cross-sectional association

Several NULISAseq targets showed significant association with the common AD risk factors age, sex, and *APOE* ε4 carrier status (Additional file 2: Figure S1). To account for the potential confounding effect of these risk factors, we utilized linear mixed models, to evaluate the adjusted significance for the cross-sectional association between NULISAseq targets and neuroimaging biomarkers.

**Fig. 2:**
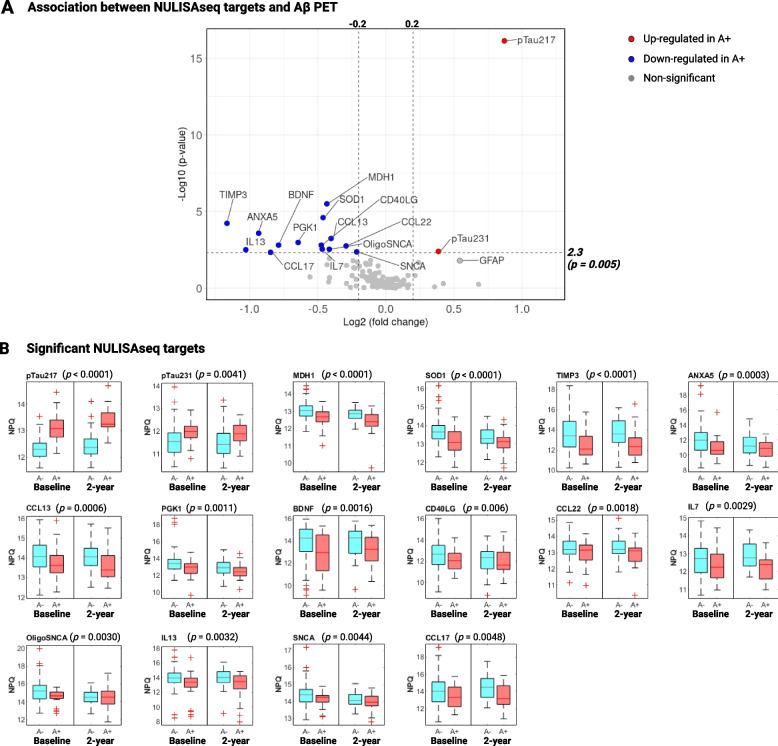
Cross-sectional association of NULISAseq targets with amyloid pathology (A). **A** Volcano plot of -log10 (*p*-value) versus log2(fold change) comparing biomarker abundances (NPQ) in samples from A + participants (n = 49) vs. A- controls (n = 127). Significant targets are shown in red (higher in A +) or blue (lower in A +) circles. Grey circles represent non-significant targets. NPQ (NULISA Protein Quantification) represents the log2-transformation of normalized target counts. **B** Boxplot distributions of significant NULISAseq targets, separated by A status and visit. P-values on top of the boxplots were for the whole data combining both visits and were determined using linear mixed models (random intercepts) with NPQs as the dependent variable, visit-specific A status as the independent variables, adjusting for covariates age, sex, and *APOE* ε4 carrier status. Significance determination was based on *p*-value < 0.005, corresponding to ~ 8% FDR

A total of 16 targets showed significant association with Aβ pathology, as determined by Aβ PET, according to *p*-value < 0.005, corresponding to approximately 8% FDR (Fig. 2A). Figure 2B illustrates boxplot distributions of significant targets at baseline and the 2-year visit. As stated above, NULISAseq plasma p-tau217 demonstrated superior diagnostic accuracy in detecting Aβ PET positivity, achieving AUCs of 0.905 (95% CI: 0.841–0.969) and 0.922 (95% CI: 0.825–0.972) at the baseline and 2-year visits, respectively, when utilized as the sole predictor. Incorporating the risk factors age, sex, and *APOE* ε4 carrier status raised the AUCs to 0.930 (95% CI: 0.878–0.983) and 0.938 (95% CI: 0.856–0.979), respectively. On average, A + participants exhibited an 82.8% elevation in plasma p-tau217 levels compared with A- controls. NULISAseq p-tau231 also exhibited significant association. However, the AUCs and fold increases were inferior to plasma p-tau217. The AUCs were 0.718 (95% CI: 0.615–0.822) in the baseline and 0.698 (95% CI: 0.556–0.821) in the 2-year samples based on biomarker-only models, which increased to 0.808 (95% CI: 0.717–0.899) and 0.794 (95% CI: 0.652–0.888) respectively, with the inclusion of the risk factors. An overall 30.7% increase was observed comparing p-tau231 levels in A + participants to those in A- controls. Moreover, GFAP showed high univariate association prior to adjusting for common risk factors, with Wilcoxon rank-sum *p*-values of 0.0002 for the baseline and 0.006 for the 2-year cohort. However, GFAP showed a strong association with age and *APOE* ε4 carrier status (Additional file 2: Figure S1B and S1C), and its risk factor-adjusted significance weakened to a *p*-value of 0.016. The fold increase of GFAP in A + vs. A- participants was 45.7%. It distinguished A + from A- participants with AUCs of 0.732 (95% CI: 0.640–0.825) and 0.715 (95% CI: 0.569–0.830) in the baseline and 2-year cohorts based on biomarker-only models, and 0.808 (95% CI: 0.717–0.899) and 0.815 (95% CI: 0.668–0.926), respectively, after adjusting for common risk factors.

Contrarily, the other targets that showed significant associations with Aβ pathology demonstrated decreased protein levels in the A + versus A- participants (Fig. 2A and B). Metalloproteinase inhibitor 3 (TIMP3), a metalloprotease inhibitor involved in regulating proteostasis [57, 58], exhibited the most substantial decrease in protein levels, with a 60%-fold decrease in A + vs. A- individuals. TIMP3 distinguished A + and A- participants with AUCs of 0.711 (95%CI: 0.596–0.814) and 0.739 (95%CI: 0.574–0.859) for the baseline and 2-year visit cohorts when used as the sole predictor. The inclusion of common risk factors improved the AUCs to 0.850 (95% CI: 0.718–0.923) and 0.882 (95% CI: 0.757–0.946). Malate dehydrogenase subunit 1, MDH1, also emerged as one of the top significant proteins, with the risk factor-adjusted *p*-value < 0.0001, and decreased at an average of 26% in A + participants. BDNF, a neurotrophic factor with pivotal roles in regulating synaptic plasticity and neuronal survival, showed an overall 42% reduction in A + participants.

Six cytokines—IL7, IL13, CD40LG, CCL13, CCL17, and CCL22—were significantly associated with Aβ pathology, supporting the involvement of immune response and inflammation in AD. Additional significant targets included superoxide dismutase 1 (SOD1), phosphoglycerate kinase 1 (PGK1), annexin A5 (ANXA5), and NULISAseq targets for soluble α-synuclein (SNCA) and oligomeric α-synuclein (OligoSNCA).

#### Longitudinal association

**Fig. 3:**
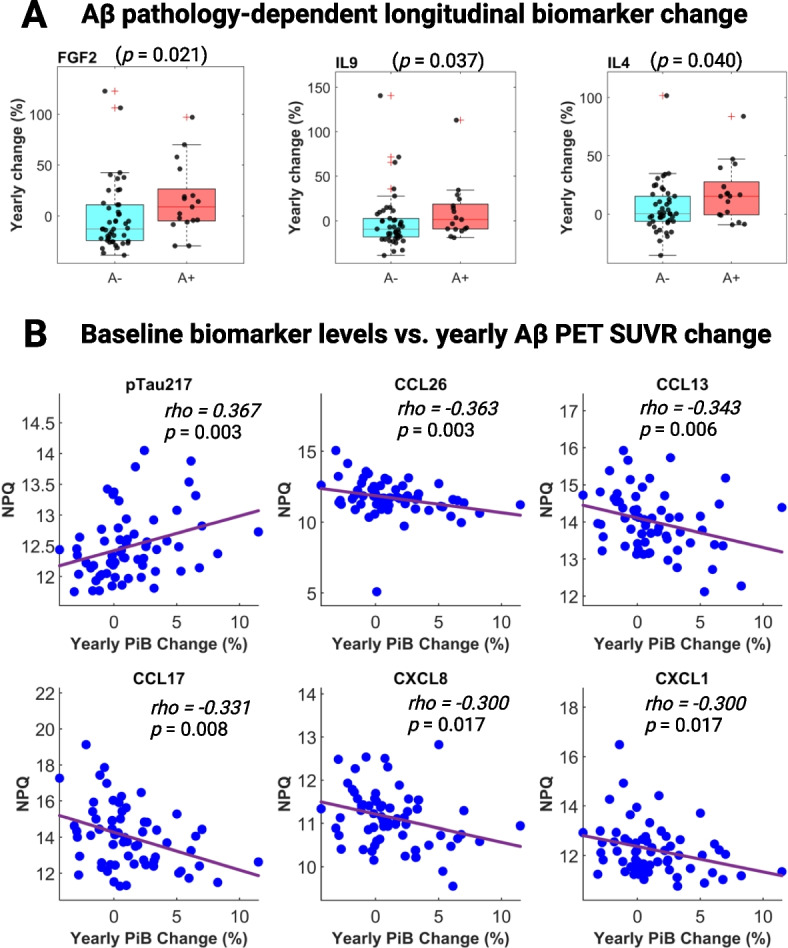
Longitudinal association between NULISAseq targets and amyloid pathology (A). **A** Boxplots illustrating the distribution of yearly biomarker abundance change by A status. *P*-values were based on two-sided Wilcoxon rank-sum tests. **B** Scatterplots for the correlation between yearly longitudinal Aβ PET SUVR change and baseline biomarker levels. The strength of the correlation was assessed based on Spearman’s ranks. Purple lines indicated the least square regression lines

We then investigated the longitudinal relationship between NULISAseq targets and Aβ pathology. Three cytokines – FGF2, IL4, and IL9 – exhibited Aβ PET-dependent yearly percentage changes, with Wilcoxon rank-sum test *p*-values of 0.02, 0.04, and 0.04, respectively (Fig. 3A). The median yearly percentage changes were 9.1%, 1.5%, and 15.4% in A + individuals, compared to -12.8%, -9.5%, and 0.4% in A- participants, respectively, for FGF2, IL4, and IL9. This means that while the cytokine levels increased over time in A + individuals, large decreases were recorded for A- participants.

Next, we explored the influence of baseline biomarker levels on the progression of Aβ pathology, defined as the yearly percentage change in Aβ PET SUVR. Apart from p-tau217, five chemokines – CCL26, CCL17, CCL13, CXCL1, and CXCL8 – demonstrated significant associations (Fig. 3B). Higher baseline levels of p-tau217 were associated with more robust increases in Aβ PET SUVR, with Spearman *rho* of 0.367 (*p* = 0.003). On the contrary, elevated baseline levels of all five chemokines were linked with a smaller Aβ PET SUVR increase, with *rho* of -0.363 (*p* = 0.004) for CCL26, -0.343 (*p* = 0.006) for CCL13, -0.331 (*p* = 0.008) for CCL17, -0.300 (*p* = 0.017) for CXCL18, and -0.300 (*p* = 0.017) for CXCL1. Given that a slower increase in Aβ PET SUVR changes is likely indicative of a more favorable prognostic outcome, these findings suggest that higher levels of these chemokines may confer a protective role in recruiting immune cells to attenuate the accumulation of Aβ plaques. Consistent with this, all five chemokines were lower in abundance in A + participants, albeit only CCL13 and CCL17 passed the significance cutoff.

We further tested the association of plasma biomarker longitudinal changes with Aβ PET SUVR changes. Seven NULISAseq targets, namely IL5, p-tau217, Aβ38, PGF, CCL2, IL4, and VEGFD, showed strong correlations (Additional file 2: Figure S2). The changes of all seven targets were positively correlated with Aβ PET SUVR changes, suggesting that upward changes of these targets over time might be correlated with more severe Aβ pathology.

### Association of NULISAseq targets with tau pathology (T)

#### Cross-sectional association

**Fig. 4:**
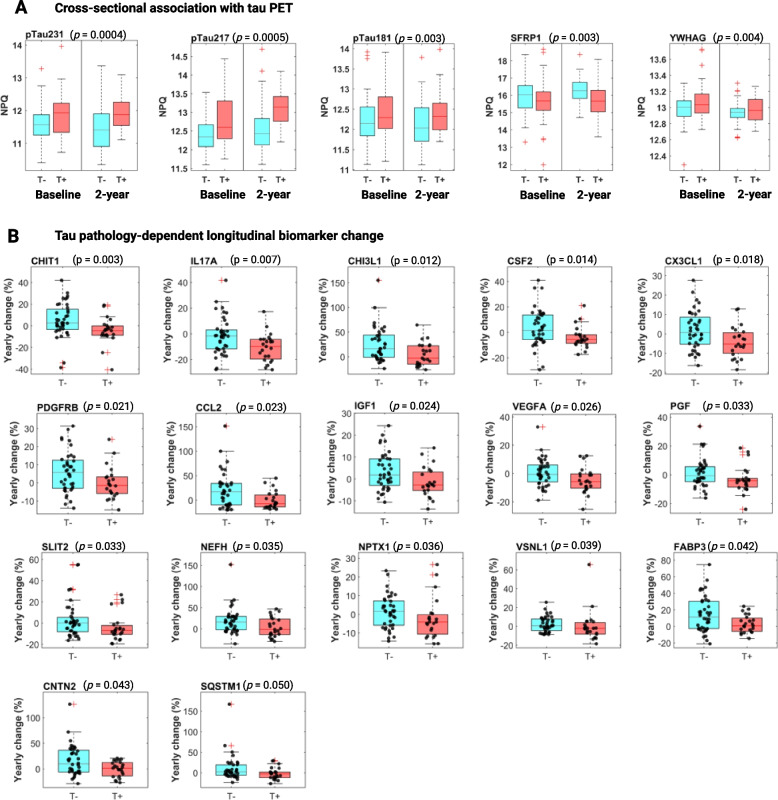
Association of NULISAseq targets with tau pathology (T). **A** Boxplots of NULISAseq targets with significant cross-sectional associations with T status, separated by T status and visit. *P*-values on top of the boxplots were for the whole data combining both visits and were determined using linear mixed models (random intercepts) with NPQs as the dependent variable, visit-specific T status as the independent variables, adjusting for covariates age, sex and *APOE* ε4 carrier status. Significance determination was based on *p*-value < 0.005, corresponding to ~ 9% FDR. **B** Boxplots illustrating the distribution of yearly biomarker abundance change by T status. *P*-values were based on two-sided Wilcoxon rank-sum tests

Five NULISAseq targets displayed significant associations with tau PET positivity according to *p*-value cutoff of 0.005, corresponding to FDR of 9%, after adjusting for age, sex and *APOE* ε4 carrier status (Fig. 4A). The three top significant targets were p-tau species, namely p-tau231 (*p* = 0.0004), p-tau217 (*p* = 0.0005), and p-tau181 (*p* = 0.003). Secreted frizzled-related protein 1 (SFRP1), a Wnt signaling modulator [59], and 14–3-3 protein gamma (YWHAG) were the other significant targets, with *p*-values of 0.003 and 0.004, respectively. All except SFRP1 were increased in T + participants. Average fold increases of 29%, 36%, 20%, and 5% in T + participants compared with T- controls were observed for p-tau231, p-tau217, p-tau181, and YWHAG, respectively. SFRP1, on the other hand, was decreased at an average of 27%. Among these five targets, p-tau217 had the highest diagnostic accuracy in detecting abnormal tau pathology, with AUCs of 0.652 (95% CI: 0.518–0.765) for the baseline cohort and 0.797 (95% CI: 0.660–0.888) for the 2-year cohort. This was followed by p-tau231, which had AUCs of 0.651 (95%CI: 0.522–0.759) and 0.705 (95% CI: 0.560–0.816), respectively. The inclusion of age, sex, and *APOE* ε4 carrier status only slightly improved the AUCs (Additional file 2: Figure S3). Both p-tau217 and p-tau231 showed better diagnostic accuracies in the 2-year cohort, consistent with the expectation that tau pathology worsens over time and that agreement between the plasma and neuroimaging biomarkers improves with disease progression.

As indicated in Table 1, there was a higher proportion of A + individuals among those who were T + . To test whether the Aβ PET status contributed to the observed association between these significant targets and tau PET status, we evaluated the association by including Aβ PET status in the linear mixed models. All remained significant except for p-tau217, which was trending towards significance (*p* = 0.068) after adjusting for the effect of Aβ PET status. The *p*-values after adjusting for Aβ PET status were 0.005, 0.003, 0.005, and 0.001 for p-tau231, p-tau181, SFRP1, and YWHAG, respectively.

#### Longitudinal association

A total of 17 targets displayed significant tau pathology-dependent longitudinal changes according to the Wilcoxon rank-sum *p*-value < 0.05 (Fig. 4B). Chitotriosidase-1 (CHIT1), a known indicator of microglial activation [60, 61], emerged as the top significant target (*p* = 0.003). Its levels showed slight increases in T- participants, with a median yearly change of 2.7%. T + participants, on the contrary, exhibited a median yearly decrease of 4.4%. Similarly, CHI3L1 (YKL-40), a biomarker for reactive astrogliosis, also exhibited an increase (median yearly change of 16.3%) in T- participants but a decrease in T + individuals (median yearly change of -2.8%).

PGF, PDGFRB, and VEGFA, important players in maintaining cerebrovascular integrity, also showed tau pathology-dependent longitudinal changes. All three exhibited a declining trend over time in T + participants (median yearly change: PGF, -4.1%; PDGFRB, -1.5%; VEGFA, -5.7%), contrasting with either stable or increased levels observed in T- individuals (median yearly change: PGF, -0.6%; PDGFRB, 5.8%; VEGFA, -0.6%). These findings suggest that tau pathology may be linked to the deterioration of vascular structure. NPTX1, a biomarker of excitatory synaptic pathology, similarly displayed a decreasing trend in T + participants (median -4.1%/year), in contrast to a slight upward change in T- controls (median 1.6%/year). Additional targets with tau pathology-dependent longitudinal changes included four cytokines (CCL2, CSF2, IL17A, and CX3CL1), proteins involved in synaptic and neuronal dysfunction (VSNL1, CNTN2, and FABP3), IGF1, SLIT2, NEFH, and SQSTM1.

Baseline levels of three NULISAseq targets, interleukin-12 (IL12p70), interferon gamma (IFNG), and RuvB-like 2 (RUVBL2), were significantly associated with tau PET changes between the two visits (Additional file 2: Figure S4A). High baseline levels of IL12p70 and IFNG, two presumptive pro-inflammatory cytokines, were associated with faster progression of tau pathology, as determined by a more pronounced increase in tau PET composite, with *rho* of 0.288 (*p* = 0.022) and 0.269 (*p* = 0.033), respectively. The opposite relationship was recorded for RUVBL2, an AAA-type ATPase involved in regulating pro-inflammatory response. A higher baseline level of RUVBL2 was associated with a smaller increase in tau pathology, with a rho of -0.253 (*p* = 0.046).

The longitudinal change of seven NULISAseq targets correlated significantly with tau PET SUVR change (Additional file 2: Figure S4B). Interestingly, the list included three tau targets, all of which showed positive associations, namely MAPT (t-tau; *rho* = 0.348; *p* = 0.005), p-tau217 (*rho* = 0.293; *p* = 0.020) and p-tau181 (*rho* = 0.251; *p* = 0.047). Other targets on the list included SOD1 (*rho* = 0.359; *p* = 0.004), interleukin-6 receptor subunit alpha (IL6R; *rho* = 0.292; *p* = 0.020), granulocyte–macrophage colony-stimulating factor (CSF2; *rho* = 0.280; *p* = 0.027), and CD40 ligand (CD40LG; *rho* = -0.262; *p* = 0.038).

### Association of NULISAseq targets with neurodegeneration (N)

#### Cross-sectional association

**Fig. 5:**
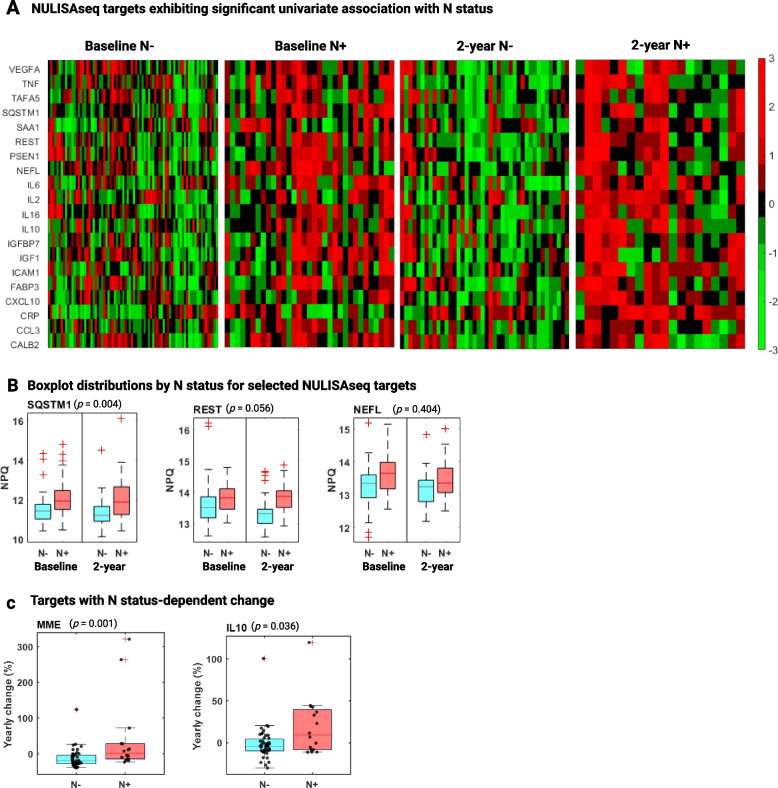
Association of NULISAseq targets with neurodegeneration (N). **A** Heatmaps illustrating the abundance levels of NULISAseq with significant univariate associations with N status (unadjusted for covariates). The NPQ values were standardized for each protein target using z-scores. **B** Boxplots of selected NULISAseq targets, separated by N status and visit. *P*-values on top of the boxplots were for the whole data combining both visits and were determined using linear mixed models (random intercepts) with NPQs as the dependent variable, visit-specific N status as the independent variables, adjusting for covariates age, sex, and *APOE* ε4 carrier status. **C** Boxplots illustrating the distribution of yearly biomarker abundance change by N status. P-values were based on two-sided Wilcoxon rank-sum tests

Twenty NULISAseq targets exhibited significant associations with N status when assessed using univariate analysis with dichotomous outcomes (N- vs. N +) or Spearman correlations with MRI-determined cortical thickness (*p*-value < 0.005, corresponding to ~ 5% FDR) without adjusting for the effects of common risk factors. The list of significant targets included NEFL, a classical biomarker for neurodegeneration, eight cytokines (IL2, IL6, IL10, IL16, TNF, CCL3, CXCL10, and TAFA5), proteins previously linked to synaptic and neuronal network defects (CALB2, FABP3, and REST), proteins involved in regulating proteostasis (PSEN1, and SQSTM1), proteins involved in acute-phase response (CRP, SAA1 and SAA2), and ICAM1 and VEGFA, both critical for maintaining cerebrovascular integrity. As depicted in the heatmap in Fig. 5A, these targets exhibited a consistent trend in both the baseline and 2-year visit samples, with all targets upregulated in N + individuals compared with N- controls. The repressor element-1 silencing transcription factor (REST), a zinc finger transcription factor with potential neuroprotective function [62], was one of the top significant targets, with *p*-values of 0.004 and 0.0008 and AUCs of 0.671 (95% CI 0.561–0.769) and 0.766 (95% CI 0.600–0.883) for the baseline and 2-year visit, respectively (Fig. 5B). NEFL showed a strong association in the baseline samples (*p* = 0.003) but not in the 2-year visit samples (*p* = 0.126) (Fig. 5B).

However, after adjusting for age, sex, and *APOE* ε4 carrier status, the associations weakened for most of these targets, with sequestosome 1 (SQSTM1) being the only target retaining a *p*-value < 0.005. SQSTM1 exhibited strong association with N status in both baseline and 2-year cohorts, with Wilcoxon rank-sum *p*-values of 0.0001 and 0.006, respectively (Fig. 5B). It distinguished N + from N- participants with accuracies of 0.729 (95% CI 0.606–0.829) at baseline and 0.713 (95% CI 0.548 -0.854) at the 2-year visit (Fig. 5B). The inclusion of common risk factors (age, sex, and *APOE* ε4 carrier status) improved the accuracies to 0.776 (95% CI 0.674–0.860) and 0.848 (95% CI 0.703 -0.933).

#### Longitudinal association

Neprilysin (MME) and interleukin 10 (IL10) demonstrated neurodegeneration-dependent abundance changes, with increases in N + participants (median change/year: MME, 1.7%; IL10, 9.4%) and decreases in N- individuals (median change/year: MME, 19.2%; IL10, 3.8%) (Fig. 5C).

Baseline levels of five NULISAseq targets – KLK6 (kallikrein related peptidase 6), CCL11, AGRN (agrin), tumor necrosis factor (TNF), and PGK1 – were significantly associated with cortical thickness change, all showing positive correlations, suggesting higher levels of these targets may be linked with a slower rate of neurodegeneration (Additional file 2: Figure S5A). The longitudinal change of five targets – PTN, YWHAZ (14–3-3 protein zeta/delta), GOT1 (glutamic-oxaloacetic transaminase 1), NRGN, Aβ42, and SNCA (oligomer) – was significantly correlated with cortical thickness change (Additional file 2: Figure S5B). Among them, Aβ42 exhibited a positive correlation with cortical thickness change, with *rho* of 0.264 (*p* = 0.037), suggesting that a decrease in Aβ42 levels is associated with more severe neurodegeneration, i.e., a decrease in cortical thickness. Conversely, changes in the other four targets were negatively associated with cortical thickness change, with *rho* of -0.335 (*p* = 0.008), -0.291 (*p* = 0.021), -0.290 (*p* = 0.022), -0.284 (*p* = 0.025), for PTN, YWHAZ, GOT1, and NRGN, respectively.

## Discussion

In this study, we have demonstrated the feasibility of concurrent immunoassay-based analysis of 116 protein markers in blood to provide diagnostic and prognostic information in preclinical AD. Our results identified several novel inflammation, synaptic, and vascular markers in blood significantly associated with brain Aβ, tau, and neurodegeneration burden at baseline and at the two-year follow-up. These were not limited to markers such as p-tau217, p-tau231, p-tau181, and GFAP, the elevation of which have consistently shown strong associations with brain Aβ and/or tau load, but included novel protein targets that inform about the disease state of the individual in different pathological stages across the biological AD continuum. Importantly, this is the first time several of these protein targets have shown validated technical and clinical biomarker potential in blood. These included the cerebrovascular markers ICAM1, VCAM1, PDGFRB, PGF, VEGFA, VEGFD, synaptic marker NPTX1, and glial markers CHIT1 and CHI3L1 (YKL-40).

Concurrent measurement of a large number of protein analytes presents technical challenges that most available immunoassay platforms struggle to address. Problems such as reagent cross-activity and the dynamic range of target analyte abundance impede the multiplexing capacity of immunoassays. Technological breakthroughs, including antibody arrays, proximity ligation assay (PLA), proximity extension assay (PEA), microsphere bead capture technology by Luminex, and slow off-rate modified aptamer assay (SOMAscan), have enabled the simultaneous measurement of hundreds to thousands of plasma proteins [63]. Among them, PEA-based Olink and SOMAscan stand out for their multianalyte measurement capacities, capable of measuring thousands of proteins in a single assay. A number of studies have utilized these proteomic platforms for AD biomarker research, leading to the identification of several emerging AD biomarkers and biomarker panels [64–67].

The NULISA technology, which is built as an advancement of the PLA technique, integrates multiple mechanisms to enhance the performance of PLA, including a proprietary sequential immunocomplex capture and release mechanism for background reduction, next-generation sequencing-based signal readout, and fine-tuning the ratio of unconjugated "cold" antibodies to DNA-conjugated “hot” antibodies to mitigate sequencing reads of high-abundant proteins. This provides the proteomic platform the capability to detect hundreds of protein biomarkers with attomolar sensitivity and ultrabroad dynamic range [34]. The high detectability rate and low detection limits for the various protein targets in this study support this. Compared to the highly multiplex Olink and SOMAscan platforms, which are designed to measure a large number of proteins for discovery applications, the NULISAseq CNS panel offers more targeted measurements of established Alzheimer’s Disease biomarkers and emerging biomarkers with known associations with neurodegenerative diseases.

The strong correlation and comparable diagnostic accuracies in the head-to-head comparisons with Simoa assays indicate that both techniques measure equivalent pools of the protein targets available in the blood. It is worth mentioning that the exceptional correlation with the Simoa ALZPath assay could be due to the two assays using the same p-tau217 monoclonal antibody.

The high performance of plasma p-tau217, p-tau231, and GFAP to identify abnormal Aβ PET scans in this mostly cognitively normal cohort is corroborated by findings from several recent studies based on results from other analytical platforms [9, 68–72]. Importantly, we identified biomarkers with decreased levels in A + participants, akin to plasma Aβ42 and Aβ42/40, indicating reduced availability in blood with progressive bran Aβ pathology. The decreases in TIMP3 are consistent with previously reported lower TIMP3 levels in AD patients [73]. TIMP3 also promotes brain Aβ production via inhibiting α-secretase cleavage of the amyloid precursor protein [74]. Reduction in MDH1 levels supports previous reports on the involvement of altered energy metabolism in late-onset AD [75, 76] while BDNF has been implicated in a protective role against Aβ peptides-induced neurotoxicity [77]. Several inflammatory cytokines were among the significantly down-regulated biomarkers, consistent with published literature that linked multiple inflammatory cytokines to Aβ pathology in AD cases and those resilient to AD [78–80]. FGF2 gene transfer reversed hippocampal function and cognitive decline in mouse models [81]. Similarly, beneficial effects of IL4 have been reported in animal models [81].

Plasma p-tau217, p-tau231, and p-tau181 were the leading markers to identify abnormal tau-PET scans. However, accounting for Aβ PET status in the combined A and T positivity analysis suggested that the results were partly explained by the strong association of these markers with Aβ pathology. This could mean that the tau forms containing these phosphorylation sites become available in blood in the early phases of Aβ plaque pathology. Aside from blood-based tau markers, YWHAG [82–84] and SFRP1 [85] showed strong associations with AD. The reduction in SFRP1 levels might be explained by its direct binding to Aβ plaques [86]. Importantly, the existing evidence from these markers has been built in CSF and brain tissue samples. Here, we extend these findings to blood.

The reactive astrogliosis marker CHI3L1 (YKL-40) and the microglia activation marker CHIT1 have repeatedly been shown to be associated with tau pathology; however, the evidence base has only been built using CSF samples [6, 87, 88]. In our study, both proteins exhibited tau pathology-dependent longitudinal changes, increasing in T- participants but decreasing in T + participants who were also Aβ-positive. These observations suggest that Aβ pathology may trigger early activation of the brain's immune system to mitigate damage, but this response may plateau or decrease as more downstream pathology, such as tau pathology, becomes apparent. Alternatively, lower glial activation in response to amyloid and tau pathology may reflect the resilience of pathologically burdened but cognitively preserved individuals [78, 89]. Tau pathology-dependent longitudinal changes were also observed in the vascular markers PGF, PDGFRB, and VEGFA, and the synaptic marker NPTX1, The biomarker potential of all these biomarkers has been demonstrated in CSF [21, 90–96]. Translation of these prior findings to plasma indicates that the molecular processes in AD involving these markers are reflected in the bloodstream, expanding the repertoire of blood-based indicators of brain pathophysiological changes.

Our study identified several plasma biomarkers with strong associations with neurodegeneration assessed based on cortical thickness, including NEFL, which is a proven general marker of neuronal injury [97, 98]. Not surprisingly, several cytokines, including IL2, IL6, IL10, IL16, TNF, CCL3, CXCL10, and TAFA5, also showed significant associations with neurodegeneration, reinforcing the close relationship between neuroinflammation and neurodegenerative processes [99, 100].

Two vascular proteins, ICAM1 and VEGFA, were on the significant list, consistent with the expected involvement of neurovascular dysfunction in neuroinflammation and neurodegeneration [101]. ICAM1 is a transmembrane glycoprotein expressed in multiple cell types and plays a key role in maintaining the blood brain barrier (BBB) [102]. Its expression is induced by neuroinflammation, leading to increased leukocyte transmigration across the BBB, a key event in the pathogenesis of various brain diseases, including AD [103–105]. Consistent with this, our study observed elevated ICAM1 levels in participants with neurodegeneration, aligning with previous research [18, 20]. VEGFs have complex associations with neurological diseases, exhibiting both neuroprotective and neuro-destructive potentials [24, 25, 106]. We observed elevated VEGFA levels in N + participants and a faster decline in VEGFA levels in T + participants, suggesting a potential staging effect. Interestingly, a recent study showed that a low level of VEGFA, measured with assays from Meso Scale Discovery, was associated with accelerated neocortical tau accumulation in preclinical A + participants in the Harvard Aging Brain Study [107].

Synaptic and neuronal network dysfunction, along with aberrant proteostasis, represent two of the eight pathological hallmarks of neurodegenerative diseases [35]. In alignment with this, we found significant associations between neurodegeneration, three synaptic/network proteins (CALB2, FABP3, and REST), and two proteostatic regulators (PSEN1 and SQSTM1). Notably, among all targets with significant association with neurodegeneration, only SQSTM1 withstood corrections for age, sex, and *APOE* ε4 genotype, revealing it as a potentially novel neurodegeneration biomarker for AD, with respect to cortical thickness. SQSTM1, a scaffold protein with a critical role in macroautophagy, has been previously linked to several neurodegenerative diseases, including AD [108–110].

IL10 and MME were the top hits with differential longitudinal changes in N + vs. N- participants. Several studies support their roles as markers of neurodegeneration status. For example, an animal model study suggested that the mechanisms of action of IL-10 as an inflammatory response might be through the activation of microglia, which leads to IL-6 activation and abnormal phosphorylation of tau [111]. MME is an integral membrane-bound metallopeptidase (MMP) and one of the key enzymes involved in Aβ degradation [112]. MMPs have been found to exhibit dual roles in AD pathogenesis. On the one hand, they can reduce the amount of Aβ deposits by degrading Aβ peptides [113, 114]. On the other hand, their levels can be induced by Aβ, potentially leading to brain parenchymal destruction [115, 116].

Interestingly, while a number of cytokines (IL2, IL6, IL10, IL16, TNF, CCL3, CXCL10, and TAFA5) were increased in participants with neurodegeneration, several cytokines (IL7, IL13, CD40LG, CCL13, CCL17, and CCL22) were found to be decreased in participants with Aβ pathology. Additionally, higher baseline levels of several chemokines (CCL26, CCL17, CCL13, CXCL1, and CXCL8) were significantly associated with slower progression of Aβ pathology. Given that most participants with Aβ pathology were cognitively normal, and neurodegeneration is presumed to occur at a later stage than the early phase of Aβ pathology, our results support the biphasic roles of neuroinflammation, with protective effects in the early stages and potentially detrimental effects in the later stages. These findings are in line with the recognized multifaceted impact of neuroinflammation on AD pathogenesis [117, 118].

Our study identified several biomarkers exhibiting pathology-dependent longitudinal changes. Specifically, FGF2, IL4, and IL9 showed Aβ pathology-dependent changes; several biomarkers associated with neuroinflammation, synaptic function, and cerebrovascular integrity demonstrated tau pathology-dependent changes; and MME and IL10 exhibited neurodegeneration-dependent changes. These findings may enhance our understanding of the natural history of AD, allowing for better staging of the disease. They can also aid in its diagnosis and prognosis, guide the development of therapeutic interventions to slow disease progression, and monitor the efficacy of treatments.

Strengths of this study include (i) technical validation of the new NULISA platform; (ii) direct comparison of the clinical performances of biomarkers measured using NULISA assays vs. with Simoa assays; (iii) focus on a population-based cohort to provide information closer to the real world than most clinical research-based cohorts; (iv) emphasis on predominantly cognitively normal participants with emerging pathological phenotypes, to test the sensitivity of the NULISA platform to these incipient changes; (v) availability of paired neuroimaging measures of Aβ, tau, and neurodegeneration, making it possible to identify inflammatory, vascular and synaptic markers associated with abnormal changes in different biologically defined disease stages; and (vi) repeated neuroimaging evaluations and blood collection over a two-year interval, allowing to examine biomarker changes within that timeframe.

Limitations include the lack of validation in larger and more diverse cohorts. Our cohort primarily consists of mostly non-Hispanic White participants, despite being representative of the catchment area. More diverse cohorts will be needed to determine whether our findings are transferable to the general population. Additionally, since the majority of participants were cognitively normal at both visits, our study does not allow for the examination of the association between NULISAseq biomarkers and cognitive function throughout the disease continuum. Lifestyle factors and comorbidities can significantly influence blood biomarkers, as recognized by recent review papers [119, 120]. However, due to the small sample size, we could not incorporate their impact into our analysis. Furthermore, the significance of longitudinal changes was based on short-term follow-up. Cohorts with longer-term follow-up, particularly those tracking clinical outcomes such as cognitive decline and the progression of AD pathology, are needed to further evaluate the clinical utility of these biomarkers.

## Conclusions

Together, this targeted proteomic study has established that results from the NULISA platform are equivalent to those from Simoa HDX. Additionally, the strong multiplexing capabilities of NULISA allowed for the evaluation of dozens of verified and putative protein biomarkers in a longitudinal preclinical AD cohort. We identified several neuroinflammation, synaptic, and vascular markers that have been previously linked to AD, but their measurement in plasma was hitherto not established. Our findings, therefore, pave the way for independent validation of these plasma markers to enable their widespread use for diagnostic, prognostic, and monitoring.

## Supplementary Information

Additional file 1: List of biomarkers included in the NULISAseq CNS panel.

Additional file 2: Supplementary Figure S1 to S5.

## Data Availability

De-identified, cohort-level data will be shared at the request of verified investigators to replicate procedures and results reported in this article. Data transfer agreements in accordance with US legislation and the decisions of the University of Pittsburgh’s Institutional Review Board, which covers the jurisdiction of the MYHAT-NI study, may need to be established.

## Abbreviations

Aβ: Amyloid-beta
AD: Alzheimer’s disease
ANXA5: Annexin A5
AGRN: Agrin
AUC: Area under Curve
BBB: Blood brain barrier
CD40LG: CD40 ligand
CDR: Clinical dementia rating
CHI3L1: Chitinase-3 like-protein-1
CHIT1: Chitotriosidase-1
CNS: Central nervous system
CNTN2: Contactin 2
CSF: Cerebrospinal fluid
CSF2: Granulocyte-macrophage colony-stimulating factor
CV: Coefficient of variation
FDR: False discovery rate
GOT1: Glutamic-oxaloacetic transaminase 1
IC: Internal control
ICAM1: Intercellular adhesion molecule 1
IFNG: Interferon gamma
IL10: Interleukin 10
IL12p70: Interleukin-12 (IL12p70)
IL6R: Interleukin-6 receptor subunit alpha
IQR: Interquartile range
IPC: Inter-plate control
KLK6: Kallikrein related peptidase 6
LOD: Limit of detection
MCI: Mild cognitive impairment
MDH1: Malate dehydrogenase subunit 1
MME: Neprilysin
MMSE: Mini-Mental State Examination
MRI: Magnetic resonance imaging
MYHAT: Monongahela Youghiogheny Healthy Aging Team
MYHAT -NI: Monongahela Youghiogheny Healthy Aging Team-Neuroimaging
NEFH: Neurofilament heavy chain
NEFL: Neurofilament light chain
NPQ: NULISA protein quantification
NPTX1: Neuronal pentraxin-1
NRGN: Neurogranin
NULISA: NUcleic acid-linked Immuno-Sandwich Assay
NULISAseq: NULISA with next-generation sequencing readout
OligoSNCA: Oligomeric α-synuclein
PEA: Proximity extension assay
PET: Positron emission tomography
PiB: Pittsburgh compound-B
PDGFRB: Soluble platelet-derived growth factor receptor β
PGK1: Phosphoglycerate kinase 1
PLA: Proximity ligation assay
p-tau: Phosphorylated tau
pTDP43-409: TDP43 phosphorylated at Ser409
PTN: Pleiotrophin
QC: Quality control
ROC: Receiver operating characteristic
ROIs: Regions of interest
RUVBL2: RuvB-like 2
SD: Standard deviation
SFRP1: Secreted frizzled-related protein 1
Simoa: Single-molecule array
SNCA: α-Synuclein
SOD1: Superoxide dismutase 1
SQSTM1: Sequestosome 1
SUVR: Standardized uptake value ratio
TIMP3: Metalloproteinase inhibitor 3
TNF: Tumor necrosis factor
UCHL1: Ubiquitin C-terminal hydrolase L1
VCAM1: Vascular cell adhesion molecule 1
VEGF: Vascular endothelial growth factor
YWHAG: 14-3–3 Protein gamma
YWHAZ: 14-3–3 Protein zeta/delta
